# The Effects of Smart Home Interface Touch Button Design Features on Performance among Young and Senior Users

**DOI:** 10.3390/ijerph19042391

**Published:** 2022-02-18

**Authors:** Na Yu, Ziwei Ouyang, Hehe Wang, Da Tao, Liang Jing

**Affiliations:** 1 College of Furnishings and Industrial Design, Nanjing Forestry University, Nanjing 210037, China; chris7251@163.com; 2 Co-Innovation Center of Efficient Processing and Utilization of Forest Resources, Nanjing Forestry University, Nanjing 210037, China; 3 College of Art and Design, Nanjing Forestry University, Nanjing 210037, China; ouyzw0418@163.com; 4 Institute of Human Factors and Ergonomics, College of Mechatronics and Control Engineering, Shenzhen University, Shenzhen 518060, China; taoda@szu.edu.cn; 5 User Experience Testing Center, China Household Electric Appliance Research Institute, Beijing 100053, China; liangj@cheari.com

**Keywords:** smart home, interface design, touch button, user performance

## Abstract

Touch technology-based smart homes have become increasingly prevalent, as they can help people with independent daily life, especially for the elderly. The aim of this study was to investigate the effects of button features (i.e., button size, graphics/text ratio, and icon style) in smart home interfaces on user performance across two age groups. Participants in the young group (*n* = 15) and senior group (*n* = 15) completed a clicking task. Button size ranged from 10 mm to 25 mm with 5 mm increments. The three levels of graphics/text ratio were 3:1, 1:1, and 1:3, while icon style was either flat or skeuomorphic. Results showed that button size and graphics/text ratio had significant effects on user performance in both groups, whereas icon style only had an effect in the senior group. It was observed that the elderly were fond of buttons with a larger size of 20 mm with larger texts and skeuomorphic icons, whereas the young preferred a button size of 15 mm with equal-sized graphics and text. These results may help to improve the accessibility and usability of smart home interface design.

## 1. Introduction

Aging has become an important public concern and received increasing attention around the world. It is estimated that the proportion of the elderly aged 65 years and more will reach 16% of the world population by 2050 [1]. Previous studies have found that, among people aged 65 years and more, more than half have vision problems, one-third have hearing problems, and nearly two-fifths have movement problems [2]. With the aging process, the elderly usually experience a gradual decline in health, thus needing assistive devices or caregivers to help with their daily lives. With the rapid development of technologies in recent years, the application of 5G technology, artificial intelligence (AI), and the Internet of things (IOT) has endowed traditional home furnishing and equipment with intelligent characteristics, leading to the concept of a smart home [3]. For most of the elderly, long-term home care appears inevitable, yet unaffordable for them. The emergence of smart homes can be a good solution to this problem. Smart homes not only enable the elderly to live a convenient and safe life, but they can also be an affordable and sustainable solution for long-term home care [4].

Smart homes can assist the elderly with their independent daily life. They can be especially beneficial for individuals with motor control disabilities. Previous research has shown that the elderly are willing to accept life changes and advantages such as emergency assistance, fall prevention and detection, and medicine reminders from smart home devices [5,6,7]. However, there is still a long way to go for the commercialization and use of smart homes. In many studies, although the word “elderly” was used in the abstract and the title of the paper, the actual experiment was carried out on young people, whereas research on middle-aged and elderly people is relatively scarce [8]. Consequently, it is necessary to carry out research for different ages and truly include the elderly population. The development of this kind of research can provide a reference for smart home design based on the performance of different age groups.

A touchscreen interface is the most direct and common way of interacting with smart home devices, which entails the need for universal design. The main reason for the success of touchscreen interfaces is that they allow direct input and are a more intuitive and accessible way to support human–computer interaction [9]. However, users still encounter many problems when using touchscreens, such as finger occlusion and individual variation [10,11,12], and the design characteristics of the interface affect user performance [13,14,15,16]. To solve these limitations, the interface design of smart home control should fully consider factors that may affect the use of the devices. Moreover, efforts should be made to develop intuitive visual user interfaces for consumers, and these user interfaces should be evaluated frequently using quality of Experience (QoE) metrics [17]. The corresponding solutions to the usability problems of touchscreen interface design were summarized in the early stage, and it was found that the solutions mostly involve the reasonable design of icons, text, interface layout, information display, text prompt, size, and location.

The purpose of this study was, therefore, to investigate the effects of touch button design features of smart home interfaces on user performance and perceptions by individuals of different ages, by taking button size, graphic/text ratio, and icon style as examples. While the majority of previous studies focused on small touchscreen devices, this study simulated a smart home terminal interface with a large touchscreen device. Results from this study could help improve the usability of smart home interfaces and guide design for users of different ages.

## 2. Related Work

### 2.1. Button Size

Earlier studies believed that a larger button size led to better performance [18,19], especially for freehand interaction [20]. However, button sizes should be kept optimal minimal because of the limited screen space in many cases. Several international standards have been developed to indicate button size recommendations. ANSI/HFES 100-2007 recommends a minimum button size of 9.5 mm, with no modifications larger than 22 mm [21]. ISO 9241-9 [22] recommends a button size of 22–23 mm (the breadth of the distal finger joint of a 95th percentile male). Other researchers believed that a button with a size of 19.05 mm and a gap of 6 mm is best [23]. Moreover, researchers found that, when the button size was 17.5 mm and larger (e.g., 19.05 mm and 20 mm), better user performance could be generated under different levels of button spacing [24,25,26]. Thus, there is no consensus on button size among these standards and previous studies. In addition, previous studies have suggested that button design should be evaluated with various user groups and situations. Chen [27] reported that user performance was better at a button size of 20 mm for the healthy group, while the disabled group had a continuously improved user performance as button size increased. Yueh [28] found that, compared with young people, the elderly preferred larger buttons with a side length of 20 mm when using touchscreens. Jin [25] found that, for the elderly with normal finger movement, a button size of 16.51 mm was best, whereas, for the elderly with low finger flexibility, a button size of at least 19.05 mm was required. Other studies considered posture. When the button size was less than 20 mm, the user performance in a standing posture was worse than that when sitting, while there was no significant difference in user performance when the button size was larger [13]. Despite more and more research being published on touchscreen use performance and related standards, studies on touchscreen use in smart home terminal interfaces are still lacking.

### 2.2. Graphic/Text Ratio

Previous studies indicated that graphic symbols can reduce the complexity of interface and improve the efficiency of information transmission. Compared with text messages, participants can more quickly and more accurately identify graphic information [29,30,31]. Meanwhile, other researchers held the view that screen interfaces with “graphics + text” have better user performance than those with “text” or “graphics” only in terms of task completion time and accuracy rate [32]. A few studies examined graphics/text ratio in web design. Lin et al. [33] found that a web page with a graphics/text ratio between 3:1 and 1:1 would be clear and easy to use, a ratio of 1:1 seemed to have the most realistic look, and a ratio of over 3:1 looked too fancy. Although previous research has suggested that the combination of graphics and text in the button should be more universal to improve legibility of the interface, few studies have examined how various ratios of graphics to text affect user performance in smart home terminal interfaces.

### 2.3. Icon Style

Icons are important graphical elements with the potential to communicate, directly affecting the quality of the interaction and the user experience [34]. Icon style is also an important button design factor that contributes to users’ feelings. It was found that participants performed better when clicking buttons with flat icons than those with skeuomorphic icons [35]. However, others reported that a search in flat text mode and a search for flat icons were associated with higher cognitive load, while the search time of flat icons was almost twice as long as that of skeuomorphic icons [36]. The main criticism was that a flat design ignores the three-dimensional nature of human brain, which is extremely sensitive to visual cues linking interfaces to the real world. Backhaus and others conducted a study with younger and elderly participants by creating two interface versions of a smart phone operating system (flat and skeuomorph). The results indicated that the elderly showed a preference for skeuomorphic design due to its understandability, whereas younger people preferred a flat design [37]. Similarly, Cho and Blaynee [11,12] also found that the elderly preferred a skeuomorphic design, exhibiting higher satisfaction, while young people favored a flat design. These findings indicate that individuals of various ages perform differently with different icon styles.

## 3. Methods

### 3.1. Experimental Design

The experiment implemented a three-factor (4 × 3 × 2) within-subject design, including button size (i.e., 10 mm,15 mm, 20 mm, and 25 mm), graphics/text ratio (i.e., 3:1, 1:1, and 1:3), and icon style (i.e., flat and skeuomorphic) as independent variables. Button shape was unified as a fillet square, and button sizes referred to the side length of square buttons. Graphics/text ratios referred to the proportion of the graphics and text in each button, and the total area was constant. Flat icons were made up of black lines, while skeuomorphic icons were colorful solid figures.

User performance was measured using objective evaluation indices (i.e., task completion time, accuracy rate, and eye movement data including saccade times and mean fixation time) and a subjective evaluation index (i.e., user preference questionnaire). Task completion time referred to the total time spent by a participant to complete a task. Accuracy rate was calculated as the percentage of times that a participant performed correctly in a task. Eye movements were sampled using an eye tracker. Saccade times and mean fixation times were used to measure the searching efficiency and cognitive load of the participants during the tasks. User preference was assessed through a paper questionnaire which was used to investigate their most preferred button design. In the questionnaire, schematics and descriptions of the three factors at all levels with equal proportion were listed (See Appendix A), from which the participants were required to select their favorite button design for the terminal interface of a smart home.

### 3.2. Participants

This experiment recruited 34 Chinese people, with 16 in the young group and 18 in the senior group. All participants had normal or corrected-to-normal vision, and those who needed vision correction were asked to wear glasses during the experiment; none of them suffered color-blindness. They had no physical or mental impairments and were all right-handed. The participants in the young group were undergraduate students, while those in the senior group were active or retired staff from the university. The eye movement data of four participants were invalid, one from the young group and three from the senior group. Their eyelids were swollen or pulled down due to cell aging, leading to a low sampling rate.

Finally, 15 participants in the young group (seven males and eight females, mean age = 24.2 years (SD = 1.8 years)) and 15 participants in the senior group (eight males and seven females, mean age = 63.0 years (SD = 6.1 years)) were included for data analysis. The average length and width of their index fingers were 72.4 mm (SD = 6.2 mm) and 14.2 mm (SD = 1.8 mm), respectively, and their average arm length was 61.8 cm (SD = 3.5 cm). All participants had experience using smart touch screen devices. In the young group, 12 subjects had experience using smart home devices, while three subjects did not. Only one subject in the senior group had experience using smart home devices, while the other 14 subjects did not. The experiment obtained the consent of all participants.

### 3.3. Materials and Tasks

**Figure 1:**
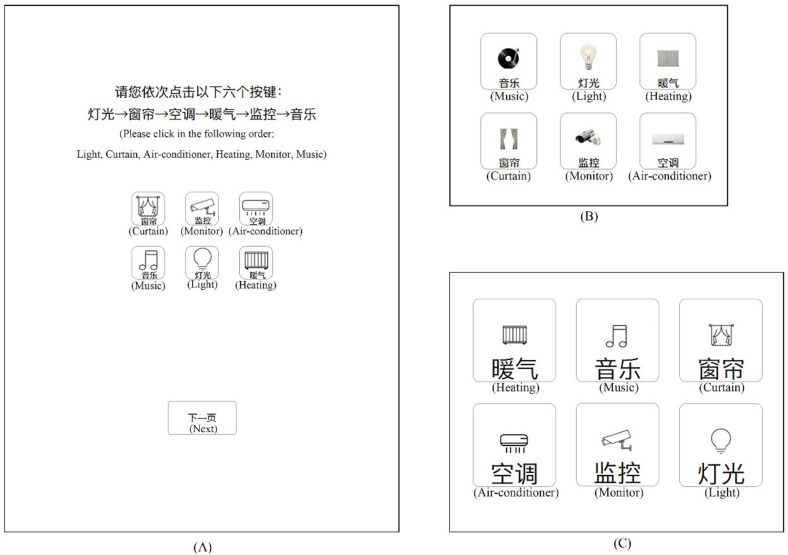
Examples of screenshot for clicking tasks of smart home touchscreen interfaces (**A**), a full page of experimental interface showing buttons with 10 mm size, graphics/text ratio of 3:1, and flat icons. (**B**), part of page showing buttons with 15 mm size, graphics/text ratio of 1:1, and skeuomorphic icons. (**C**), part of page showing buttons with 25 mm size, graphics/text ratio of 1:3, and flat icons. English words in parentheses were not shown during the experiments).

A smart home terminal interface prototype was developed with Axure (Axure Software Solution, San Diego, CA, USA) and MockingBot (Beijing Modaokeshi Technology Co., Ltd., Beijing, China). The prototype was presented on a Huawei tablet PC with an EMUI 9.1 operating system (8.4 inch screen with a resolution of 2560 × 1600 pixels). Referring to a previous study [13,27], the display screen was at a 70° angle to the desk surface. Eye movements were sampled using an eye tracker (Tobii Pro Nano, Tobii Tech., Stockholm, The Kingdom of Sweden), with a sampling rate of 60 Hz and a spatial accuracy of 0.3° or higher. The human–machine environment test cloud platform (ErgoLAB, Kingfar International Inc., Beijing, China) was also used to measure the behavior data and eye-movement data. Miller [38] found that the capacity of visual short-term memory is 7 ± 2 bits. Considering the capacity and design aesthetics, the experimental interface contained six buttons with six commonly used functions (i.e., light, curtain, air conditioner, heating, monitor, and music) in smart homes. The selection was based on a market survey and user questionnaires; icons normalized by GB/T 35417-2017 [39] were excluded in the experiment (see Figure 1 for an example). There were 24 pages in the prototype. The interface background was white, and all text was black. Texts prompting a click were shown at the top of each page, and six buttons with constant spacing of 6 mm were set below the texts. In the task, participants were required to click the six buttons in order according to the text prompt. The order of the text prompt and buttons was random. The button color was changed to yellow within 0.1 s as visual feedback after each button was pressed by the participant, before immediately returning to its original color. Data on user performance (i.e., task completion time and accuracy rate) and eye movement were recorded.

### 3.4. Procedures

This experiment was conducted in the Ergonomic Laboratory of Nanjing Forestry University. Before the experiment, participants were informed the procedure, and they could stop at any time. After providing informed consent, participants were asked to fill out a personal information questionnaire including their demographic information and physical condition. A research assistant measured the length and width of the right index finger and the length of their right arm. Then, participants were tested for their cognitive ability using the Flanker task. After adjusting the seat to the appropriate angle and height and being informed about the operation procedure, participants were asked to complete the pretests, and then the formal experiment began after the practice. The whole experiment was divided into three parts. Participants took a 3 min break after the completion of each part (eight pages and six click tasks on each page), before continuing with another part. The order of the buttons and text prompts on each page was random. Participants were required to click the buttons according to the text prompt as quickly and accurately as they could. Upon the completion of all parts, they were asked to fill out the user preference questionnaire about their button design preference. The whole experiment lasted about 20 min.

### 3.5. Data Analysis

First, to examine whether objective evaluation variables were normally distributed, the Shapiro–Wilk test was applied, while three-way repeated-measures analyses of variance (ANOVAs) were used to analyze the effects of button size, graphics/text ratio, and icon style. Post hoc tests were performed using the Bonferroni correction for multiple comparisons. Sensitivity analyses were used to adjust the analyses for gender, the length and width of the index finger, and the arm length in both groups, but no significant effect was observed. A chi-square test was performed to examine the differences in user preference between the two groups. The significance level was set at *p* < 0.05 for all statistical tests. Statistical analyses were performed using SPSS Version 22 (IBM, Armonk, NY, USA).

## 4. Results

### 4.1. Cognitive Ability

The Flanker task was used to measure the cognitive ability of all participants. The variation coefficients of reaction time and accuracy rate of each participant were calculated, all of which were less than 0.15 (Table 1). It has been suggested that a coefficient of variation greater than 0.3 indicates that the data are faulty or that the experimental variables are uncontrollable [40]. The results indicated that the dispersion of data was small, and the decision-making ability and response ability of all participants were in a normal and equal range.

### 4.2. Task Accuracy Rate

Table 2 shows the ANOVA analysis results for accuracy rate. Button size was found to have a significant effect on accuracy rate in both groups, revealing the lowest accuracy rate for a size of 10 mm, while there was no significant effect for the larger three sizes. Icon style had a significant effect in the senior group, whereby they achieved a higher accuracy rate for the skeuomorphic icons. No other significant effect was observed.

### 4.3. Task Completion Time

**Figure 2:**
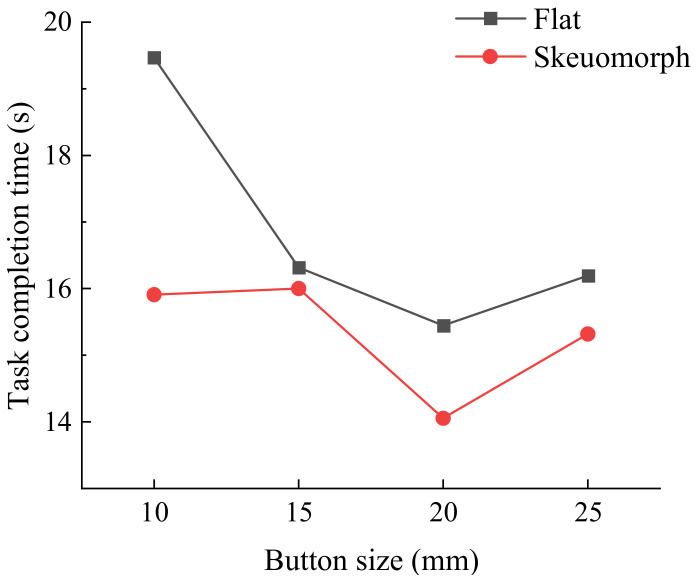
Task completion time (s) by button size and icon style for senior group.

Table 3 shows the ANOVA results for task completion time. In the young group, graphics/text ratio was found to have a significant effect on task completion time, while button size and icon style did not show any significant effect. As graphics/text ratio decreased (i.e., graphics area decreased and text area increased), the task completion time of the young participants decreased. As for the senior group, all factors had significant effects on task completion time. Both groups had a shorter task completion time for 20 mm button size, but the factor of button size in the young group had no significant effect. In the senior group, the paired comparison showed that the task completion time for 10 mm was significantly different to that for 20 mm and 25 mm. As for icon style, buttons with a skeuomorphic icon resulted in significantly faster performance than buttons with a flat icon for the senior group. For the young group, buttons with a flat icon were a little bit faster, but there was no significant effect. In the senior group, the interaction between button size and icon style was significant (F = 2.968, *p* < 0.05) (Figure 2).

### 4.4. Number of Saccades

**Figure 3:**
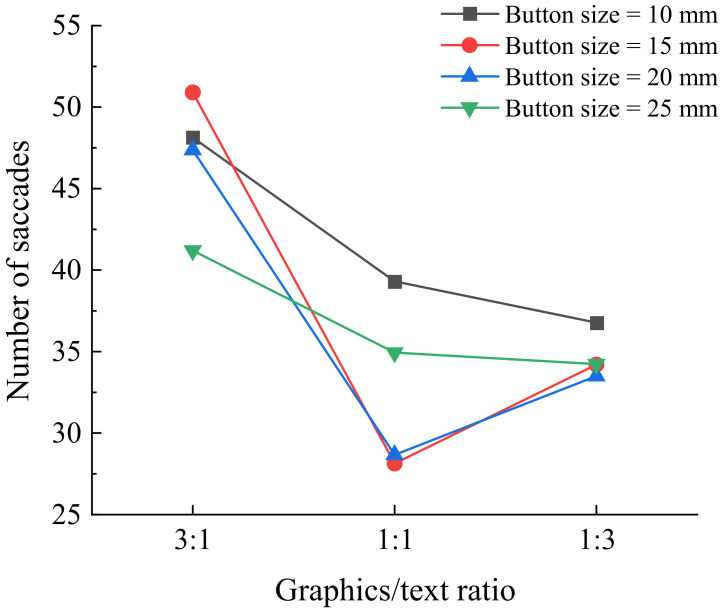
Number of saccades by graphics/text ratio and button size for young group.

Table 4 presents ANOVA results for number of saccades in eye movement data. Button size was found to have a significant effect on number of saccades for the young group but not for the senior group. The young group had the most saccades when the button size was 10 mm, which was significantly different from the other three levels in the paired comparison, while the paired comparison results of 15 mm, 20 mm, and 25 mm showed no significant difference. Graphics/text ratio showed a significant effect on number of saccades for both groups. The young participants had fewer saccades at the 1:1 graphics/text ratio, while the senior participants had fewer saccades at the 1:3 graphics/text ratio. As for icon style, no significant effect was observed. Graphics/text ratio presented a significant interaction with button size (F = 4.365, *p* < 0.001) for the young group (Figure 3).

### 4.5. Mean Fixation Time

**Figure 4:**
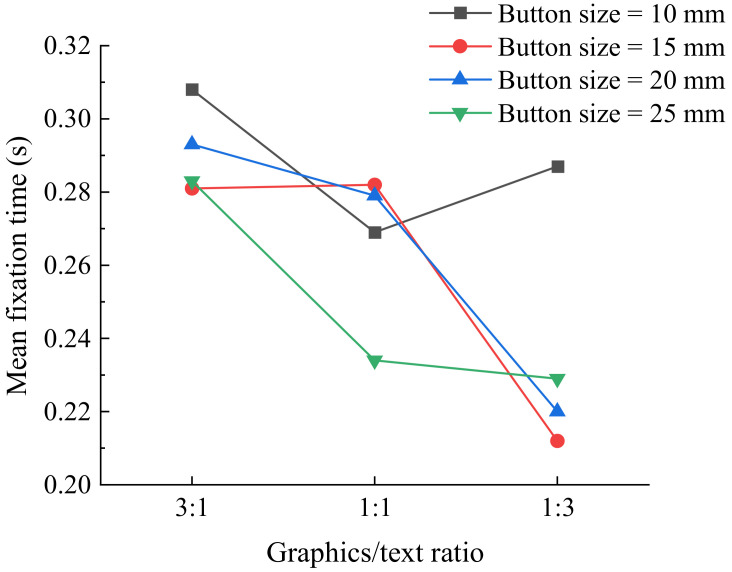
Mean fixation time (s) by graphics/text ratio and button size for senior group.

Table 5 presents the results of ANOVA for mean fixation time in eye movement data. For the young group, only button size was found to have a significant effect on mean fixation time, while there was no significant effect of the other factors. Mean fixation time plateaued at 10 mm button size in the young group, which was significantly different from the other three sizes in the paired comparison. As for the senior group, all of the factors were found to have a significant effect on mean fixation time. The mean fixation time of the senior group for the 25 mm button size and a graphics/text ratio of 1:3. In terms of icon style, the senior participants presented a significantly shorter mean fixation time for the skeuomorphic icons. A significant interaction between button size and graphics/text ratio (F = 2.738, *p* = 0.012) was observed in the senior group (Figure 4).

### 4.6. User Preference

Table 6 shows user preference in terms of button size, graphics/text ratio, and icon style. More young people preferred the 15 mm button size (46.7%) and flat icons (60%), but the difference was not significant. A majority of young participants preferred the graphics/text ratio of 1:1 (60%; χ^2^ = 6.4, *p* < 0.05), which showed a significant difference. For the senior group, more people preferred the 1:3 graphics/text ratio (53.3%), but the difference was not significant. Most senior participants preferred the 20 mm button size (80%; χ^2^ = 14.8, *p* = 0.001) and skeuomorphic icons (86.7%; χ^2^ = 8.067, *p* < 0.05), which showed a significant difference.

## 5. Discussion

The popularization of smart homes has led to an increasing number of consumers using smart home systems. To get more people to accept smart homes, especially the elderly, it is necessary to make the interface of smart homes easy to use. This research focused on the performance of users of different ages, which can effectively help designers select the design features of smart home interface buttons, so as to better improve usability and achieve a better user experience. By simulating the terminal interface of smart home and clicking tasks, the present study was conducted to examine the effects of three design characteristics (i.e., button size, graphics/text ratio, and icon style) on user performance and perceptions in two age groups. Due to aging, the user performance of senior participants was worse than that of young participants in all factors. Icon style had no significant effect on young participants, while other factors had significant effects. Graphics/text ratio had a significant interaction with button size and icon style in the young group. Young participants preferred a graphics/text ratio of 1:1 and a 15 mm button size. All factors had significant effects on the senior participants, where button size was found to interact with graphics/text ratio and icon style. They preferred a 20 mm button size, a graphics/text ratio of 1:3 (i.e., smaller graphics area and larger text area), and skeuomorphic icons.

### 5.1. Effects of Button Size

The experimental results indicate that the young group had better user performance with button sizes of 15 mm and above, while the senior group had better performance with button sizes of 20 mm and above. All participants had difficulty in recognizing the smaller 10 mm button size. This was consistent with previous studies [23,24,25,27,28]. There was almost no difference in accuracy when using 15 mm, 20 mm, and 25 mm buttons among users, especially among young people. However, according to other indicators, a 20 mm button was more suitable and more popular among users. Moreover, in the terminal interface design of a smart home, the button size cannot be increased infinitely for better usability because of the limitation of screen size. In order to maximize user performance in a limited screen, designers and engineers need to think carefully how to balance the two factors and meet more performance requirements.

### 5.2. Effects of Graphics/Text Ratio

Previous studies reported that a ratio of graphics to text between 3:1 to 1:1 would be clearer and more usable in web page design [33]. However, the participants were young people, whereas middle-aged and elderly people were not considered in the study. In this study, the elderly were taken into consideration. The results indicate that user performance of the participants in young group improved for a ratio of graphics to text of 1:1 and 1:3, while the senior participants had better user performance for a 1:3 ratio, which is inconsistent with a previous study in web design [33]. This difference was caused by the experimental materials used. Lin’s study was based on web pages and discussed the overall graphic ratio of web pages, while this study was based on a touchscreen and discussed the ratio of icons and texts in one button. In different situations, an individual’s preference will vary. The interaction between button size and graphics/text ratio showed that elderly user performance improved as button size increased up to 15 mm with a ratio of graphics to text of 1:3; alternatively, a button size of 25 mm and a graphics/text ratio of 1:1 also yielded good performance. With larger button sizes and smaller graphics/text ratios, it is obvious that texts on the button become larger and clearer so that participants can rapidly identify the button on the basis of the text. A larger text leads to better guidance, probably because, although graphics can represent things more vividly, texts can be understood intuitively. Another explanation may be that users’ fingers covered the graphics or texts on buttons of a smaller size, which made clicking tasks complicated. Consequently, buttons with larger texts may have poor appearance despite excellent user performance and high recognition efficiency.

### 5.3. Effects of Icon Style

Another important contribution of the present work to the literature is that this study provides evidence on the effects of icon style in two age groups. Significant effects of icon style on user performance were not found in the young group, indicating that young people might focus on the texts more frequently or that the effectiveness of flat design and skeuomorphic design were equal. However, the senior participants showed significant differences with respect to icon style. The skeuomorphic icons improved user performance of the elderly, indicating that icon style played an important role in the process of elderly recognition. It is also important to note that a significant interaction effect between button size and icon style was observed in the senior group. Buttons with a skeuomorphic icon resulted in a faster reaction, even for a button size of 10 mm. This suggests that icon style affects the recognition efficiency of old people, and that skeuomorphic icons facilitates understanding of the information conveyed by the button. While flat design is a style derived from minimalism, users from an older generation are not familiar with it. When users control smart home devices with operating errors, there are potential safety risks. Collectively, designers should explicitly take the familiarity and habits of older generations into account during design. Skeuomorphic icons may be more readily understandable and acceptable by the majority of individuals.

### 5.4. User Preference

Subjective preference of users is increasingly recognized in interface design [41,42], but this is largely ignored in smart interface design guidelines. User choice of products is largely based on subjective preferences; hence, it is necessary to pay attention to user perceptions and preference. In this study, participants in different age groups had different preferences for the interface design of a smart home. Young people held the view that button size did not need to be too big, with 15 mm identified as an appropriate size. They considered 10 mm buttons a bit small for a large touch screen, while some considered 20 mm to 25 mm buttons also acceptable. More than half of the young people chose a 1:1 graphics/text ratio. In addition, 60% of them preferred buttons with flat icons, but no significant difference was observed. They felt that flat icons looked simple and comfortable, as they are frequently used in many operating systems such as Windows and Mac OS. In contrast, a majority of the senior participants favored the 20 mm button size, a graphics/text ratio of 1:3, and skeuomorphic icons. These results are consistent with the objective evaluation of the experiment. For the elderly generations, large texts are very efficient for searching and controlling, especially when they encounter icons that they are not familiar with. However, skeuomorphic icons will also alleviate this issue.

### 5.5. Limitations

Only two healthy age groups were selected to participate in this study, and they were asked to complete the tasks in a seated posture. It is unknown whether user performance would be affected by people of different regions, races, and abilities, as well as by different postures and context of use. Users do not have to be sitting when controlling smart home devices; they are likely to be in standing, lying, or even walking postures. It remains to be further investigated whether these elements affect the results of this study. Second, the presence of text on the button may have affected the experimental results, especially when the effect of icon style was investigated. For participants who are used to reading text only, the text may attract their attention, whereby the icon is probably ignored. Future studies could exclude factors which may affect the experimental results and confirm their effectiveness. Third, because of the low complexity of tasks in the experiment, participants could easily complete them with high satisfaction. In practice, however, users are expected to carry out a series of searching and clicking steps if they want to perform a certain function when controlling smart home devices. This difference may have affected the results of this study. Lastly, although an operation timeout in smart home devices poses some safety concerns, the wrong operation is more dangerous for vulnerable groups. For such studies, the degrees of safety, easiness, and satisfaction, combined with a subjective evaluation, can be applied to verify user performance. Older people may have a long reaction time; hence, the validity of the task completion time index requires further study. Future work could focus on a usability evaluation of smart home terminal interfaces and establish a multi-assessment system. In addition, the touchscreen is a complex technology, whereby screen resolution, pixel size and spacing, color restoration and contrast, and other factors can also affect the user’s interactive performance [43]. Although these elements were consistent in this work, these influencing factors are also worthy of investigation, and subsequent studies can focus on these aspects.

## 6. Conclusions

This study investigated the effect of button size, graphics/text ratio, and icon style on user performance in young and senior groups. While performing clicking tasks on a simulated smart home system, the search performance and eye movement were measured. The user preference could be verified by questionnaire and interview. There was a significant difference between the two age groups. The user performance of the senior group plateaued for a larger button size of 20 mm with larger texts and skeuomorphic icons, while the young group preferred intermediate options. Understanding how people in different ages and abilities interact with smart home interfaces can allow designers and engineers to improve usability. Furthermore, vulnerable groups should be given priority in product design and development, so that they can live an independent and comfortable life. In light of the results of this study, a rounded square button with a side length of 20 mm, a graphic and text ratio of 1:3, and skeuomorphic icons should be used in the design of functional buttons to appeal to a broader range of users. It is also worth mentioning that, although this study was conducted from the perspective of a smart home, the research results with regard to touchscreen buttons in terms of size, graphic/text ratio, and icon style can have certain reference value for other designs. In particular, with regard to the ratio of icon to text in a button, the results show that allocating more space to text on buttons can achieve better user performance.

## Figures and Tables

**Table 1:** Results of reaction time and accuracy rate on flanker tasks for all participants.

	Young Group			Senior Group		
	Mean	SD	Coefficient of Variation	Mean	SD	Coefficient of Variation
Reaction time (ms)	498.263	56.406	0.113	672.274	94.043	0.140
Accuracy rate (%)	98.5	1.5	0.016	98.4	1.5	0.015

**Table 2:** Main effects of button size, graphics/text ratio, and icon style on task accuracy rate (%).

		Young Group				Senior Group			
		Descriptive Analysis		ANOVA		Descriptive Analysis		ANOVA	
		Mean	SD	F-Values	*p*-Values	Mean	SD	F-Values	*p*-Values
Button Size	10 mm	96.111	9.026	3.541	0.015	92.963	11.443	4.784	0.003
	15 mm	98.518	5.385			96.111	8.673		
	20 mm	98.704	5.138			96.481	8.094		
	25 mm	98.703	4.490			97.777	5.698		
Graphics/Text Ratio	3:1	98.055	6.181	0.019	0.981	95.139	9.516	1.150	0.318
	1:1	97.917	7.022			95.555	9.598		
	1:3	98.055	5.792			96.805	7.263		
Icon Style	Flat	98.333	5.599	0.924	0.337	94.907	9.665	3.933	0.048
	Skeuomorph	97.685	6.995			96.759	7.898		

**Table 3:** Main effects of button size, graphics/text ratio, and icon style on task completion time (s).

		Young Group				Senior Group			
		Descriptive Analysis		ANOVA		Descriptive Analysis		ANOVA	
		Mean	SD	F-Values	*p*-Values	Mean	SD	F-Values	*p*-Values
Button Size	10 mm	10.251	1.769	2.260	0.081	17.688	5.285	8.784	<0.001
	15 mm	9.864	1.874			16.159	4.229		
	20 mm	9.613	1.736			14.750	3.382		
	25 mm	10.106	1.989			15.757	4.089		
Graphics/Text Ratio	3:1	10.632	1.904	21.009	<0.001	18.100	4.805	25.074	<0.001
	1:1	10.078	1.727			15.470	3.940		
	1:3	9.167	1.627			14.700	3.696		
Icon Style	Flat	9.861	1.795	1.105	0.294	18.856	4.528	13.902	<0.001
	Skeuomorph	10.057	1.909			15.321	4.164		

**Table 4:** Main effects of button size, graphics/text ratio, and icon style on number of saccades.

		Young Group				Senior Group			
		Descriptive Analysis		ANOVA		Descriptive Analysis		ANOVA	
		Mean	SD	F-Values	*p*-Values	Mean	SD	F-Values	*p*-Values
Button Size	10 mm	41.400	12.135	4.151	0.007	60.478	32.622	0.613	0.607
	15 mm	37.744	14.752			59.622	30.945		
	20 mm	36.511	13.273			57.800	28.884		
	25 mm	36.789	10.108			63.944	33.003		
Graphics/Text Ratio	3:1	46.900	13.828	64.032	<0.001	67.108	36.022	4.536	0.011
	1:1	32.758	8.792			59.058	29.140		
	1:3	34.675	10.267			55.217	27.290		
Icon Style	Flat	38.244	12.447	0.058	0.810	60.767	31.061	0.034	0.853
	Skeuomorph	37.978	13.135			60.156	31.728		

**Table 5:** Main effects of button size, graphics/text ratio, and icon style on mean fixation time (s).

		Young Group				Senior Group			
		Descriptive Analysis		ANOVA		Descriptive Analysis		ANOVA	
		Mean	SD	F-Values	*p*-Values	Mean	SD	F-Values	*p*-Values
Button Size	10 mm	0.258	0.043	10.671	<0.001	0.288	0.080	4.824	0.003
	15 mm	0.239	0.031			0.258	0.081		
	20 mm	0.231	0.045			0.264	0.086		
	25 mm	0.227	0.035			0.249	0.075		
Graphics/Text Ratio	3:1	0.243	0.037	1.241	0.290	0.291	0.087	16.897	<0.001
	1:1	0.237	0.038			0.266	0.074		
	1:3	0.236	0.047			0.237	0.074		
Icon Style	Flat	0.239	0.043	0.008	0.927	0.289	0.081	39.770	<0.001
	Skeuomorph	0.239	0.038			0.241	0.074		

**Table 6:** Distribution of user preference by button size, graphics/text ratio, and icon style.

	Young Group			Senior Group		
	Percentage	χ^2^	*p*-Values	Percentage	χ^2^	*p*-Values
Button Size						
10 mm	0	2.800	0.351	0	14.800	0.001
15 mm	46.7%			13.3%		
20 mm	40.0%			80.0%		
25 mm	13.3%			6.7%		
Graphics/Text Ratio						
3:1	33.3%	6.400	0.043	26.7%	2.800	0.351
1:1	60.0%			20.0%		
1:3	6.7%			53.3%		
Icon Style						
Flat	60.0%	0.600	0.607	13.3%	8.067	0.007
Skeuomorph	40.0%			86.7%		

## Data Availability

The data presented in this study are available on request from the corresponding author. The data are not publicly available due to them being used as a reference for further experiments.

## Appendix A

Preference questionnaire				
Subject No.________				
Please choose an option that best describes your answer for each question.				
1.	Which button size do you prefer most?			

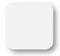
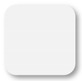
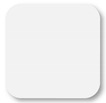
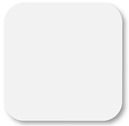
				
	10 mm	15 mm	20 mm	25 mm
2.	Which ratio of graphics to text (“light”) do you prefer most?			

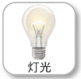
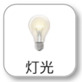
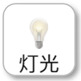
			
	3:1	1:1		1:3
3.	Which icon style do you prefer?			

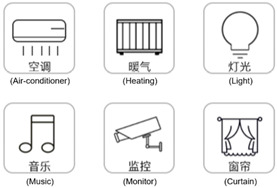
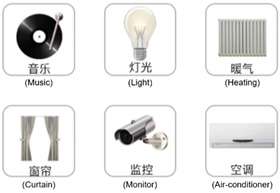
		
	Flat		Skeuomorph	
